# Limited dorsal myeloschisis with a contiguous stalk to human tail-like cutaneous appendage, associated with a lipoma of conus medullaris: A case report

**DOI:** 10.1016/j.ijscr.2020.05.021

**Published:** 2020-05-21

**Authors:** Auricelio Batista Cezar-Junior, Igor Vilela Faquini, Kauê Frank, Luiz Euripedes Almondes S. Lemos, Eduardo Vieira de Carvalho, Nivaldo S. Almeida, Hildo Rocha Cirne Azevedo-Filho

**Affiliations:** a Instituto de Medicina Integral Professor Fernando Figueira (IMIP), Recife, Brazil; b Neurosurgery Department of the Hospital da Restauração Gov. Paulo Guerra, Recife, Brazil

**Keywords:** Limited dorsal myeloschisis, Human tail, Lipoma, Cutaneous appendage

## Abstract

**Introduction:**

Limited dorsal myeloschisis (LDM) is a recently described pathological entity, characterized by a defect of the closed focal neural tube and a fibroneural pedicle connecting the cutaneous lesion to the spinal cord.

**Presentation of the case:**

This case describes a 9-month-old child with a human tail and an underlying spinal dysraphism. This was represented by LDM stalk associated with a medullary lipoma, in connection with the dorsal cutaneous appendage. We also report the therapeutic proposal for this case and its clinical outcome.

**Discussion:**

LDM is a distinctive clinicopathological presentation of a spinal dysraphism, associated with numerous anomalies, such as lipomyelomeningocele, tethered cord, lipoma, congenital heart disease and teratoma. In this case, surgical treatment for LDM consists of surgical resection of the appendage, untethering of the spinal cord and resection of conus medullaris lipoma in the same procedure.

**Conclusion:**

In this case report, we share the experience of a referral service in pediatric neurosurgery regarding clinical and radiological diagnosis, and the successful treatment of this rare type of congenital malformation. Therefore, clinicians should be aware of possible morphological variations of the skin lesion associated with LDM.

## Highlights

• Limited dorsal myeloschisis (LDM) is a milder form of myeloschisis, containing an area of incomplete closure of neural folds.
• We describe a case report of a human tail-like cutaneous appendage, associated with an intradural spinal lipoma.
• The relationship between the appendix and the LDM tract, and its treatment are discussed.
• Therefore, clinicians should be aware of possible morphological variations of the skin lesion associated with LDM.

## 1 Introduction

Limited dorsal myeloschisis (LDM) is a clinicopathological entity, described for the first time in 1993, associated with a particular case of cervical myelomeningocele [1]. It consists of a milder form of myeloschisis, without an exposed neural plate but still containing, essentially, a small and segmental area of incomplete dorsal closure of the neural folds [[2], [3], [4]]. LDM has two essential characteristics: a closed focal neural tube defect and a fibroneural pedicle, connecting the cutaneous lesion to the spinal cord [5,6]. Embryologically, there is an incomplete division of cutaneous and neural ectoderm [[5], [6], [7]]. These lesions, therefore, should be treated by untethering the stalk from the spinal cord [5,6].

The human tail is a benign congenital anomaly composed of adipose tissue, connective tissue, muscle, vessels, nerves, and mechanoreceptors. Those features of the dorsal cutaneous appendage can be distinguished from the pseudo-tail, since the latter is commonly associated with underlying spinal dysraphism, which requires special treatment [[2], [3], [4]]. We describe in this article one rare case of LDM patient with a human tail-like cutaneous appendage as an external skin manifestation, associated with an intradural spinal lipoma adjacent to the lesion, treated in the Neurosurgery department of Hospital da Restauração, Recife, Brazil. The relationship between the appendage and the LDM tract, and the treatment performed are discussed.

## 2 Case report

### 2.1 History and examination

**Fig. 1:**
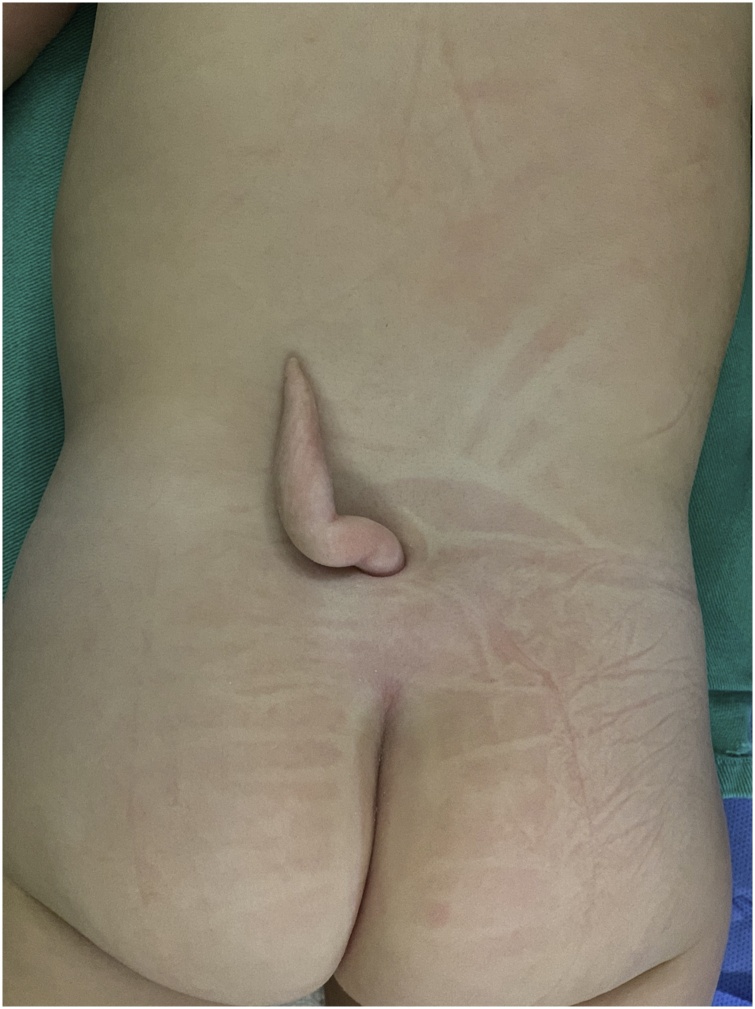
Human tail-like cutaneous appendage in the patient's lumbosacral region.

This case was reported according to the SCARE 2018 criteria [8]. The patient was a nine-month-old baby with “tail-like” structure, arising from midline of the lower back since birth. Physical examination revealed a 6 cm long appendage attached to the sacrum, like a human tail (Fig. 1). It was completely covered by seemingly normal skin on inspection. The patient and his mother had not previously used any medication. There was no family history of congenital malformation, nor psychosocial events that may have a causal relationship with this malformation. There were no apparent focal deficits on the neurological examination.

### 2.2 Initial imaging

**Fig. 2:**
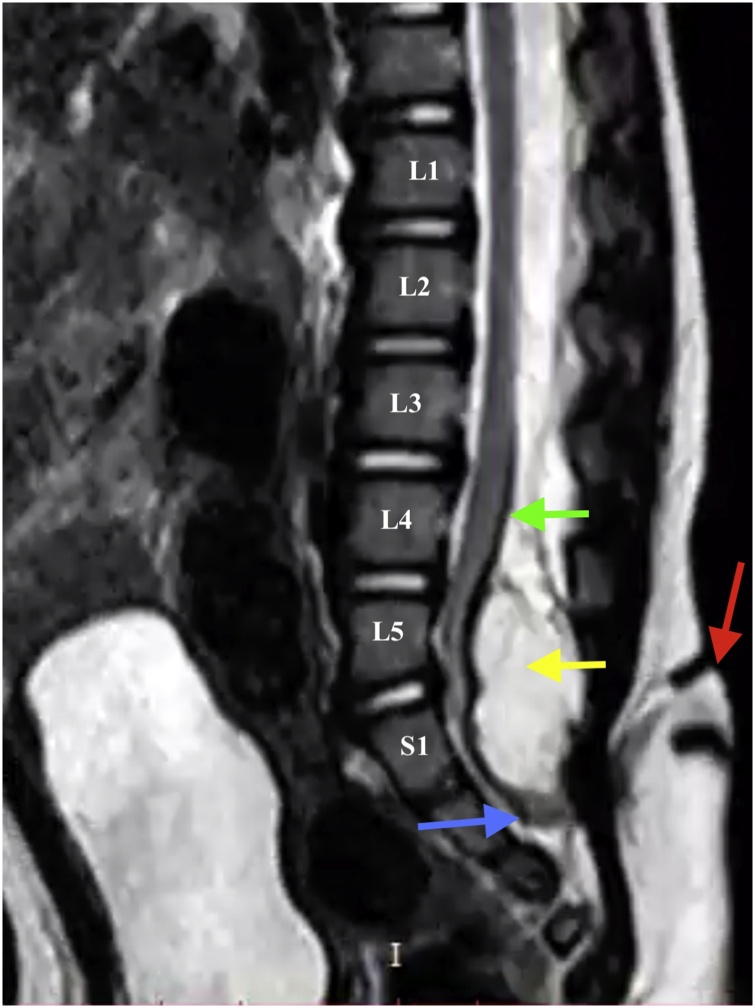
Human tail-like cutaneous appendage centered on the patient's lumbosacral region.

Magnetic resonance imaging (MRI), especially 3D-hT2WI (slice thickness, 1.25 mm) was initially performed and revealed a spina bifida at the level of L5 and along the extension of the entire sacral spine. The tail was attached to trunk at S1-S2 level. The conus medullaris had low implantation. It was possible to visualize the connection between the spinal cord and the dorsal cutaneous appendage through a fibroneural stalk. Adjacent to LDM stalk, a dorsal lipoma can be visualized in the T2 MRI (Fig. 2). Computed tomography (CT) scan was performed additionally for a better evaluation of vertebral bone structures, and the extent of laminectomy indicated to adequately expose the intra-medullary lipoma. There was an incomplete closure of the posterior spinal cord and the bony vertebral arch (lamina), in the lumbosacral region adjacent to the tail insertion.

Bladder symptoms are difficult to assess in infants. Therefore, ultrasonography was requested for early detection of renal and urinary tract anomalies. It showed upstream ureteral dilation of the right distal ureteral segment with reduced caliber, and a left kidney in a pelvic location (renal ectopia), with signs of ipsilateral vesicoureteral reflux and mild hydronephrosis. No associated anorectal malformations were seen.

### 2.3 Surgical treatment

**Fig. 3:**
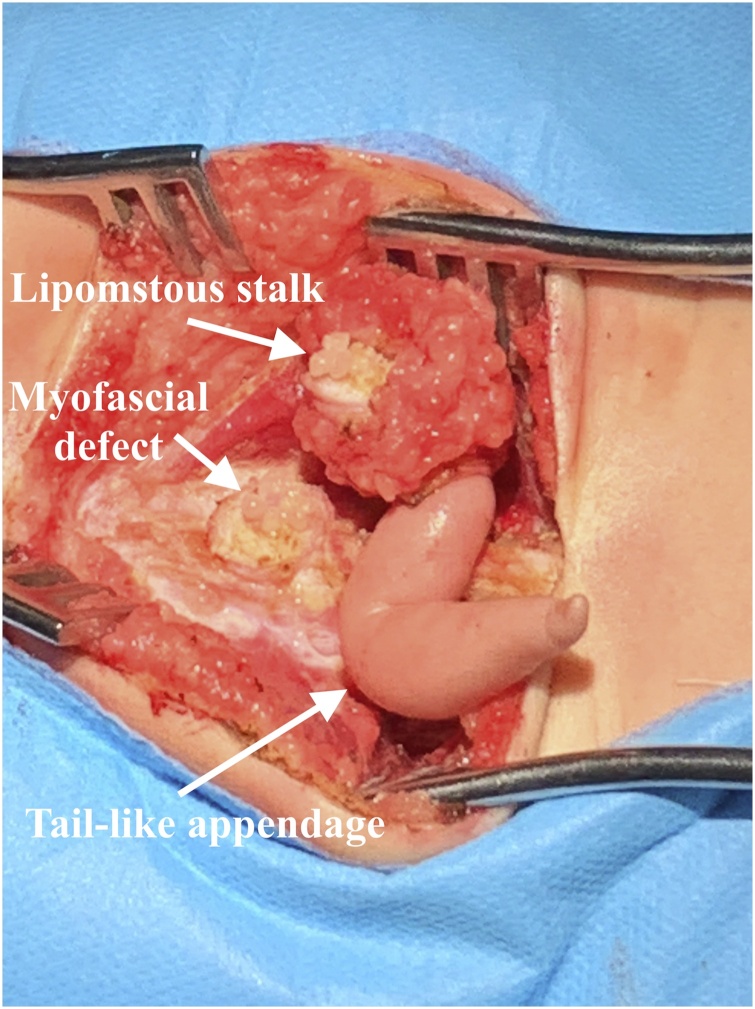
Lipomatous pedicle at the base of the appendage was dissected. The myofascial defect was located below the level of S2, which gives passage to the lipomatous pedicle.

The surgery consisted of untethering the spinal cord and disconnect the neurofibrous stalk between the spinal cord and the dorsal cutaneous appendage. Removal of the cutaneous appendage and the subtotal resection of the lipoma of conus medullaris were also performed by a pediatric neurosurgeon with large experience in surgical management of spinal dysraphisms. Adherence between nerve roots and lipoma prevented its safe gross total resection. A fibroadipose bundle continued toward the intradural space S1–S2 (Fig. 3). Identification and isolation of the thin fibroneural stalk was not possible in this case. A tight filum terminale was found intraoperatively. Therefore, surgical release of the tethered cord was performed to prevent progressive neurological symptoms related to the tethered cord syndrome. After removing the intradural segment of the lipoma, the wide dural defect was fashioned either by direct dural repair or using heterologous dural graft. Extradural space was sealed by fibrin glue, applied in the suture area (graft–dural interface). Afterward, the superficial layers were reconstructed in the usual manner. The patient remained in prone position in the postoperative period and there were no signs of CSF leak during the follow-up for the next 6 months. The patient and his family adhered to medical guidelines in the postoperative period and presented good surgical wound care.

**Fig. 4:**
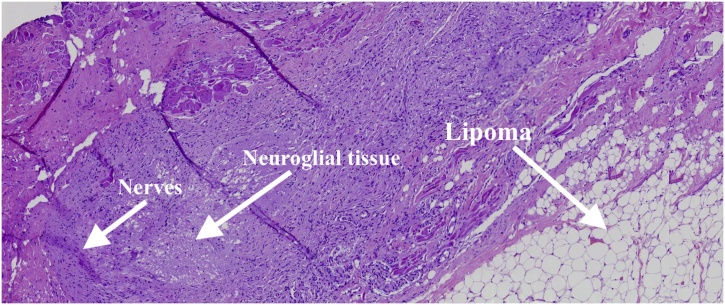
The histopathology slide of the fibroneural pedicle reveals a central lipoma, and peripherally located nerves and neuroglial tissue.

Dorsal cutaneous appendage histopathology revealed mature fibroadipose tissues covered by skin tissue. Subcutaneous lipomatous stalk consisted of peripheral nerves, lipomatous and neuroglial tissues, which were contiguous to the fibroadipose tissue of the appendage (Fig. 4). Glial fibrillary acidic protein (GFAP) was not evidenced by the immunohistochemistry of the fibroneural stalk. The patient was discharged 72 h after the operation, with no signs of CSF leak or surgical wound dehiscence, and with no neurological deficits. Her parents were quite satisfied with the surgical, aesthetic and functional results.

## 3 Discussion

Limited dorsal myeloschisis (LDM) is a distinctive clinicopathological presentation of a spinal dysraphism, associated with a dorsal midline defect, a fibroneural stalk, and a direct connection between the dorsal cutaneous lesion (e.g., human tail) and the underline spinal cord [6,4,9,10]. Furthermore, there are numerous anomalies associated with LDM, such as lipomyelomeningocele, tethered cord, lipoma, congenital heart disease and teratoma [10,11,12,13,10,14].

Lipomas of conus medullaris consist of spinal dysraphisms associated with subcutaneous adipose masses [11,15,16]. The complementary diagnosis of DLM is based on the histopathological evaluation and with the finding of neural elements, as peripheral nerve fibers and the positivity for GFAP-immunopositive neuroglial tissue in the immunohistochemical evaluation of the fibrocollagenous band [12,17,13]. In the case reported, despite evident anatomical relationship between the intra/extradural fibroneural stalk and the dorsal cutaneous appendage, GFAP was negative in the immunohistochemical evaluation of the stalk. The positivity of GFAP is difficult with conventional histopathological examination, since small islands that would demonstrate reactivity may be lost, during the cut of the stalk and the selection of small fragments to be evaluated.

Pang et al. [7] described a sample of 51 patients with DLM who were surgically treated. In 1993, the same author [1] demonstrated some diagnostic criteria for the classification of dysraphism as LDM. These criteria can be summarized as follows: a cutaneous signature of a focal area of incomplete full-thickness skin, and a fibroneural or fibrovasculoneural stalk connecting the base of the skin lesion to the underlying spinal cord. These two factors reflect a fundamental error during primary neurulation: incomplete disjunction between the skin and neural ectoderm. According to the new classification of spinal lipomas based on embryonic, proposed by Morota et al. [18], the case reported can be described as type 1 spinal lipoma. Therefore, lipoma was visible dorsal to the conus medullaris in the MRI (Fig. 3), extended caudally and connected to the subcutaneous fat and to the dorsal cutaneous appendage, through the spina bifida. In addition, spinal cord was tethered caudally. Conus medullaris was free at end of surgery, and the cord – sac ratio, which was 78% preoperatively, progressed to <30% postoperatively. Cord – sac ratio estimates the degree of freedom of motion of the spinal cord within its dural sac. According to Pang et al., [19] the low cord-sac ratio (<30%) on postoperative MRI is strongly correlated with good outcome. The cord-sac ratio was obtained by dividing the sagittal diameter of the cord by the sagittal diameter of the dural sac at the main region of the lipoma resection [19].

The surgical procedure for LDM should be performed by cosmetic removal of the appendage and untethering of the cord during the same operation [20,21,10]. In addition, in this clinical case, subtotal resection of the lipoma of conus medullaris was performed in the same sequence of procedures.

## 4 Conclusion

The current case describes a 9-month infant who had a human tail with an underlying spinal dysraphism. There are numerous reports in the literature about the existence of occult spinal dysraphism. However, an LDM stalk associated with a medullary lipoma in connection with the dorsal cutaneous appendage is a rare entity that lacks information in the literature, that can guide the best diagnostic and therapeutic management for these cases. In this case report, we share the experience of a referral service in pediatric neurosurgery regarding clinical, radiological diagnosis, and the successful treatment of this rare type of congenital malformation. Therefore, clinicians should be aware of possible morphological variations of the skin lesion associated with LDM.

## Declaration of competing interest

On behalf of all authors, the corresponding author states that there is no conflict of interest.

## Funding

No funding was received for this research.

## Ethical approval

The ethics committee of Hospital da Restauração - Recife, Brazil, conducted a review of the submission and concluded that activities described in this study do not constitute human subjects research as the projectdoes not involve identifiable private information from the patientand the subject has consented to the publication of their case. As aresult, 455 CFR part 46 does not apply.

## Consent

Consent to publish the case report was obtained from the patient's parents via the original surgical consent, which includes consent for identified publications. This report does not contain any personal in for mation that could lead to the identification of the patient.

## Author contribution

Auricelio Batista: study concept or design, data collection, data analysis or interpretation, writing the paper, revision of the content.

Igor Faquini: study concept or design, data collection, data analysis or interpretation, writing the paper.

Kaue Frank: data collection, data analysis or interpretation, writing the paper.

Luiz Euripedes: data collection, data analysis or interpretation, writing the paper.

Eduardo Vieira: data collection, data analysis or interpretation, revision of the content.

Nivaldo Sena: study concept or design, revision of the content.

Hildo Azevedo: study concept or design, revision of the content.

## Registration of research studies

Name of the registry: Research Registry

Unique identifying number or registration ID: researchregistry5518

Hyperlink to your specific registration (must be publicly accessible and will be checked): https://www.researchregistry.com/browse-theregistry#home/registrationdetails/5e9c5ad9b0afeb001b740f23/

## Guarantor

Auricelio Batista Cezar Junior.

## Provenance and peer review

Not commissioned, externally peer-reviewed.
